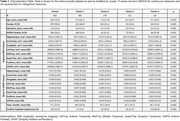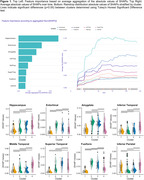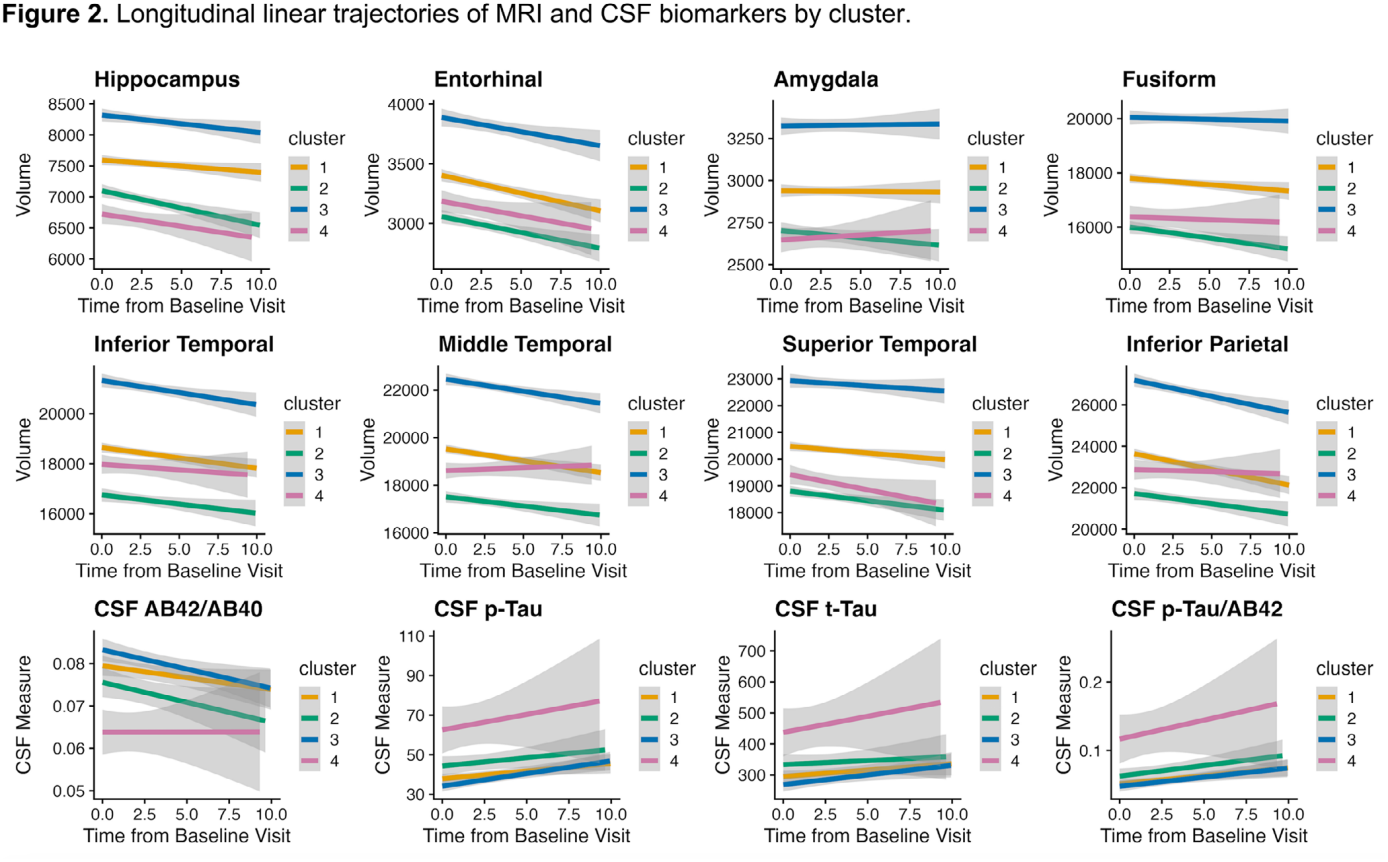# Supervised clustering of MRI measures informed by incident cognitive impairment via aggregated survival model SHAP values

**DOI:** 10.1002/alz.089947

**Published:** 2025-01-09

**Authors:** Kellen K. Petersen, Bhargav Teja Nallapu, Idris Demirsoy, Richard B. Lipton, Ellen Grober, John C. Morris, Jason J. Hassenstab, Brian A. Gordon, Ali Ezzati

**Affiliations:** ^1^ Albert Einstein College of Medicine, Bronx, NY USA; ^2^ University of California, Irvine, Irvine, CA USA; ^3^ Washington University in St. Louis, School of Medicine, St. Louis, MO USA; ^4^ Washington University St. Louis, St. Louis, MO USA; ^5^ Washington University in St. Louis School of Medicine, St. Louis, MO USA

## Abstract

**Background:**

Alzheimer’s disease (AD) is a complex heterogeneous neurodegenerative disease. Unsupervised clustering techniques have been used to identify disease subtypes, but such approaches are limited since subtypes may not directly be related to disease progression. Herein, we implement a novel supervised clustering approach that aims to identify MRI‐derived subtypes that are likely to experience incident cognitive impairment (ICI).

**Methods:**

We used data from 822 cognitively normal individuals (68.4 ± 9.5 years old) with Clinical Dementia Rating® (CDR®) of 0 at baseline, from the Knight ADRC. We performed supervised clustering of eight volumetric MRI regional measures at baseline. MRI measures were adjusted for age, sex, and education. Mean aggregated time‐dependent Shapley Additive exPlanation (SHAP) values were derived from random survival forest models with an ICI outcome based on time‐to‐conversion from a CDR=0 to CDR>0 over 10 years of follow‐up. K‐means clustering was then applied to SHAP values. Analysis of Variance was used to assess cluster differences and linear regression was used to understand longitudinal trajectories of MRI volumetrics and CSF biomarkers.

**Results:**

In the entire sample, hippocampus, entorhinal, and amygdala SHAP values were determined to be the most important MRI measures in terms of estimated contribution to the model prediction with the hippocampus differentiating from other regional measure about 24 months from baseline (Figure 1). The optimal number of clusters was determined to be four. Cluster characteristics and significant differences among them are presented in Table 1 and Figure 1. Cluster 4 is characterized by the largest mean hippocampus SHAP value with approximately 89% of participants experiencing ICI. Figure 2 shows longitudinal linear trajectories for MRI and cerebrospinal measures. Cluster 1 was the healthiest across all longitudinal biomarkers. Whereas, Cluster 4 had the smallest hippocampal volume and abnormal CSF measures both at baseline and over time.

**Conclusions:**

We implemented a novel application of SHAP values from survival models in a supervised clustering approach to identifying MRI‐subtypes with different probabilities of incident cognitive impairment. This approach could be used to elucidate the heterogeneity inherent in the relationships among AD biomarkers and longitudinal cognitive changes.